# Efficacy of Synthetic Furanones on *Listeria monocytogenes* Biofilm Formation

**DOI:** 10.3390/foods8120647

**Published:** 2019-12-05

**Authors:** Pedro Rodríguez-López, Andrea Emparanza Barrenengoa, Sergio Pascual-Sáez, Marta López Cabo

**Affiliations:** 1 Laboratory of Microbiology and Technology of Marine Products (MICROTEC), Instituto de Investigaciones Marinas (IIM-CSIC), C/Eduardo Cabello 6, 36208 Vigo, Spain; pedro.rodriguezlopez@unipr.it (P.R.-L.); andreae2000@hotmail.com (A.E.B.); pascualfeci@gmail.com (S.P.-S.); 2 Department of Food and Drug, Università di Parma, Strada del Taglio 10, 43126 Parma, Italy

**Keywords:** biofilms, food industry, food safety, furanones, *Listeria monocytogenes*

## Abstract

Furanones are analogues of acylated homoserine lactones with proven antifouling activity in both Gram-positive and Gram-negative bacteria though the interference of various quorum sensing pathways. In an attempt to find new strategies to prevent and control *Listeria monocytogenes* biofilm formation on stainless steel (SS) surfaces, different concentrations of six synthetic furanones were applied on biofilms formed by strains isolated from food, environmental, and clinical sources grown onto AISI 316 SS coupons. Among the furanones tested, (Z-)-4-Bromo-5-(bromomethylene)-2(5*H*)-furanone and 3,4-Dichloro-2(5*H*)-furanone significantly (*p* < 0.05) reduced the adhesion capacity (>1 log CFU cm^−2^) in 24 h treated biofilms. Moreover, individually conducted experiments demonstrated that (Z-)-4-Bromo-5-(bromomethylene)-2(5*H*)-furanone was able to not only significantly (*p* < 0.05) prevent *L. monocytogenes* adhesion but also to reduce the growth rate of planktonic cells up to 48 h in a dose-dependent manner. LIVE/DEAD staining followed by epifluorescence microscopy visualisation confirmed these results show an alteration of the structure of the biofilm in furanone-treated samples. Additionally, it was demonstrated that 20 µmol L^−1^ of 3,4-Dichloro-2(5*H*)-furanone dosed at 0, 24 and 96 h was able to maintain a lower level of adhered cells (>1 log CFU cm^−2^; *p* < 0.05). Since furanones do not pose a selective pressure on bacteria, these results represent an appealing novel strategy for the prevention of *L. monocytogenes* biofilm grown onto SS.

## 1. Introduction

Biofilms are considered a major issue of concern in food-related premises being the main form in which bacteria persist environmentally, potentially leading to eventual cross-contamination of foodstuffs [1]. These structures are straightforwardly defined as highly organised communities embedded in a self-produced extracellular polymeric matrix [2], providing resident bacteria an enhanced capability to endure external aggressions such as desiccation, UV radiation, or biocides [3].

Among foodborne pathogens, *Listeria monocytogenes* has gained importance due to its increasing incidence trends over the last decade [4]. In humans, especially among young, pregnant, and immunocompromised individuals, infections caused by this microorganism can provoke listeriosis, a rare but severe illness with symptoms varying from mild gastroenteritis to severe alterations to the nervous system, miscarriage, or systemic infections [5,6].

Although great advances have been made in controlling *L. monocytogenes* in the food industry, its prevalence, especially in fish and fishery ready-to-eat (RTE) products, is still remarkable [4]. Therefore, large *L. monocytogenes* outbreaks are still taking place. As a matter of example, the European Centre for Disease Control documented a multi-country *L. monocytogenes* serogroup IVb, Sequence Type 6 (ST6), outbreak from 2015 to 2017, affecting 26 people with a fatality rate of 15.4% [7]. More recently in 2017–2018, an outbreak, also associated with *L. monocytogenes* ST6, was declared in South Africa with a total 1060 cases of human listeriosis registered and a fatality rate of a 20.38% [8] in what is so far considered the largest listeriosis outbreak ever documented [9].

It has been demonstrated that in *L. monocytogenes,* mechanisms related to bacterial cell-to-cell communication, i.e., quorum sensing (QS), are responsible for many communal behaviours such as promoting initial adhesion to abiotic surfaces and subsequent biofilm formation [10]. Broadly speaking, these mechanisms are based on the self-production, releasing, and sensing of chemical messengers, the so-called autoinducers (AIs), differing in their nature depending on the bacterial species [11].

It is widely accepted that in Gram-negative species, QS is mediated through acylated homoserine lactones (HSLs), whereas in Gram-positives it is via autoinducing peptides (AIPs) [12]. In particular, *L. monocytogenes* AIP is encoded by the *agrBDCA* operon and directly affects its adherence [13] and cell invasion capacity [14]. Nevertheless, a recent study carried out by Naik, Bhangui and Bhat demonstrates that despite the lack of production of HSL-mediated system molecules, *L. monocytogenes* is able to positively respond to short-chain homoserine lactones (i.e., C_6_-HSL), promoting its biofilm-forming capacity on polystyrene plates and borosilicate glass coverslips [15].

Blocking the QS-mediated systems has been proposed as an antibiofilm strategy hindering the initial adhesion and subsequent formation of mature structures. With this aim, several furanones (analogues to HSLs) initially described in the seaweed *Delisea pulchra* and with proven antifouling capacity, have been proposed as biofilm inhibitors [16,17]. Furthermore, these compounds generally neither pose a selective pressure nor affect bacterial planktonic growth, thus limiting the possible development of antimicrobial resistances [18]. Their effects have been tested in Gram-negative species such as *Escherichia coli* [19,20] and *Salmonella enterica* [19,21,22] and Gram-positives such as *Bacillus subtilis* [11,23]. Nevertheless, to the best of the authors’ knowledge, to date, no studies dealing with the effects of furanones in biofilm formation in *L. monocytogenes* have been carried out.

In food-related environments, furanones have been described as an effective strategy to inhibit the growth of food spoilers such as *Pseudomonas aeruginosa* [24] or pathogens of high relevance in aquaculture, such as those belonging to *Vibrio* species [25]. Additionally, furanones have been postulated to be used as food additives. With this regard the European Food Safety Authority Panel on Food Contact Materials, Enzymes, Flavourings and Processing Aids, have determined that the use of these compounds, does not raise safety concerns in terms of toxicity [26,27].

Considering all the above, the purpose of the present study is to quantitatively determine the effects that commercially available furanones have on the formation of *L. monocytogenes* biofilms grown on stainless steel (SS) coupons.

## 2. Materials and Methods

### 2.1. Origin of L. monocytogenes Isolates

All isolates used are summarised in Table 1. *L. monocytogenes* used in this study were from three different origins: environmental, clinical and foodstuffs.

Environmental isolates came from surfaces in food-related premises A1, E1 and F1 were isolated in a previous survey [28], whereas G1 strain was kindly provided by Dr. Luisa Brito [29].

Regarding isolates from food sources, L1 and L8 were isolated from RTE fish products, L34 was isolated from a frozen panga fillet in a previous study [30] and L12 was isolated from a Halibut fillet as follows: 25 g of product was mixed with 225 mL of buffered peptone water (BPW; Cultimed, Barcelona, Spain) and processed with a stomacher masticator (IUL Instruments, Barcelona, Spain) for 1 min. From this sample, serial dilutions (up to 10^3^) were made in BPW, 1 mL of each solution was spread onto two (500 µL each) PALCAM (Liofilchem, Roseto degli Abruzzi, Italy) and incubated for 24–48 h at 37 °C. Following this, five *L. monocytogenes* presumptive colonies (grey–green colour with a black lowered centre and a surrounding black halo) were picked and subcultured on Trypticase Soy Agar (TSA; Cultimed, Barcelona, Spain) and incubated for 24 h at 37 °C to ensure purity. Next, the identification of the isolate was performed via 16S rRNA gene amplification, sequencing and electropherogram analysis following a protocol described previously [28].

Finally, clinical isolates were kindly provided by Dr. Maximiliano Álvarez (Hospital Álvaro Cunqueiro, Vigo, Spain) from cases of human listeriosis. These were detected from blood culture using the BD BACTEC^TM^ system (BD, NJ, USA) followed by isolation onto non-selective media, i.e., blood agar and chocolate agar (bioMérieux; Marcy-l’Étoile, France).

Stock cultures were kept at −80 °C in sterile brain–heart infusion broth (BHI; Biolife, Milan, Italy) containing 50% glycerol 1:1 (*v v^−1^*) mixed. Working cultures were kept at −20 °C in trypticase soy broth (TSB; Cultimed, Barcelona, Spain) containing 50% glycerol 1:1 (*v v^−1^*) mixed.

### 2.2. L. monocytogenes RAPD-PCR Subtyping

To ascertain the genetic relationships among the isolates tested, a random-amplified polymorphic DNA (RAPD)—PCR was chosen. Genomic DNA (gDNA) was obtained from liquid cultures following a protocol described by Vázquez-Sánchez et al. [31]. Resulting gDNA was used for RAPD using primers UBC155 (5′-CTGGCGGCTG-3′), HLWL85 (5′-ACAACTGCTC-3′), OMP-01 (5′-GTTGGTGGCT-3′) and DAF4 (5′-CGGCAGCGCC-3′) following the conditions described by Wulff et al. [32]. PCRs were performed in a MyCycler^TM^ Thermal Cycler (Bio-Rad, Hercules, CA, USA) and resulting bands were resolved in a 1.5% agarose gel stained with Red Safe (iNtRON Biotechnology, Sangdaewon-dong, South Korea) using Hyperladder 50 bp (Bioline, Singapore) as a molecular size marker and visualised using a GelDoc2000 Apparatus equipped with Quantity One, Version 4.5 software (Bio-Rad, Hercules, CA, USA).

Similarity factors based on the dice coefficient, cluster analysis by the Unweighted Pair Group Method with Arithmetic mean (UPGMA) system and dendrograms (tolerance 1%, optimisation 0.5%) were obtained using BioNumerics 7 software (Applied Maths NV, Sint-Martens-Latem, Belgium).

### 2.3. L. monocytogenes Biofilms Growth

To reactivate the isolates, 100 µL of working cultures was transferred to 5 mL sterile TSB and incubated overnight at 37 °C and subcultured twice to ensure proper reactivation.

Inocula preparation was performed by adjusting the Abs_700_ of each culture to 0.1 ± 0.001 in sterile phosphate buffer saline (PBS) using a 3000 Series scanning spectrophotometer (Cecil Instruments, Cambridge, England). This corresponded to a cellular density of approximately 10^8^ CFU mL^−1^, according to previous calibrations. This suspension was further diluted 1:1000 in sterile TSB to reach a final concentration of 10^4^ CFU mL^−1^.

In all cases, biofilms were grown onto 10 × 10 × 1 mm AISI 316 SS coupons (Comevisa, Vigo, Spain). Coupon preparation included individual washing with industrial soap (Sutter Wash, Sutter Ibérica, S.A., Madrid, Spain) to remove grease residues, rinsing with tap water, followed by a final wash with deionised water and finally autoclaved at 121 °C for 20 min. Following this, coupons were individually placed into a 24-flat bottomed well plate (Falcon, Corning, NY, USA), and each well was inoculated with 1 mL of each *L. monocytogenes* isolate. Plates were incubated at 25 °C for 2 h to allow primary adhesion and then in constant shaking at 100 rpm 25 °C.

Before any sampling was performed, samples (SS coupons) were collected aseptically and immersed for 10 s in 1 mL sterile PBS to remove loosely attached cells.

### 2.4. Plate Count Assays

#### 2.4.1. Adhered Viable and Cultivable (AVC) Cells Quantification

In all cases, AVC determination was carried as follows: at each sampling time and once PBS washing was carried out, AVC were harvested from 3 different samples (i.e., stainless steel coupons) by thoroughly swabbing of each coupon surface with two sterile cotton swabs (Deltalab, Rubí, Spain) moistened in sterile BPW. Every two swabs were pooled together in 2 mL of sterile BPW and vigorously vortexed for 1 min to release cells. Resulting suspensions were serially diluted in BPW and spread onto trypticase soy agar (TSA; Cultimed, Barcelona, Spain) plates and incubated at 37 °C for 24–30 h. Results were expressed as the mean value in log CFU cm^−2^.

In these assays, the accepted limit of detection was 25 CFU in the plate corresponding to the lowest dilution, which corresponded to 1.70 log CFU cm^−2^ [33].

#### 2.4.2. Planktonic Viable Cells Quantification

In case a quantification of planktonic cells was required, i.e., to ascertain the viability of the culture after the addition of furanones (see Section 2.5.2. for further detail), 100 µL of the liquid culture corresponding to each sample (i.e., the culture in which each individual culture was immersed) was serially diluted in sterile BPW and plated onto TSA plates. Following this, plates were incubated at 37 °C for 24 h, and the resulting colonies were counted. Results were expressed in log CFU mL^−1^.

As in AVC quantification, the accepted limit of detection was 25 CFU in the plate corresponding to the lowest dilution.

### 2.5. Biofilm Inhibition Assays

All furanones used in this study were purchased from Sigma–Aldrich (Table 2). In all cases, they were dissolved in absolute ethanol to a concentration of 1 mol L^−1^ and kept at −20 °C.

#### 2.5.1. Furanones’ Effects Screening

For the first screening of biofilm inhibition assays, furanones were added to the inoculum in each well to obtain a final furanone concentration of 2 mmol L^−1^ in Fn1 and 20 mmol L^−1^ for the rest of the furanones. The concentrations used in this and subsequent experiments were based on those utilised in previous published studies involving furanones with proven inhibitory effects on bacterial biofilms [19,23]. For controls, the same volume of absolute ethanol used in the corresponding test coupons was added to each well. Sampling of furanone-treated biofilms (*n* = 3 coupons for each concentration) and controls (*n* = 3 coupons) was performed at 24 h, as described in Section 2.4.1.

After carrying out this first experiment, two additional experiments were performed to assess the individual effects on *L. monocytogenes* L34 biofilm formation of Fn1 and Fn3 dosed at different concentrations and the effects of Fn3 dosed at different biofilm ages.

#### 2.5.2. Effects of Fn1 and Fn3 Dosed at Different Concentrations on L34 Biofilm Formation and Planktonic Cell Growth

The first additional experiment was intended to assess the effects of Fn1 and Fn3 dosed at different concentrations. For this, Fn1 was added to the mL of L34 culture at *t* = 0 h to achieve a final concentration of 0.05 mmol L^−1^, 0.2 mmol L^−1^ and 2 mmol L^−1^. Similarly, Fn3 was added to achieve a final concentration of 0.5 mmol L^−1^, 2 mmol L^−1^ and 20 mmol L^−1^. In both cases, for control samples and equal volume of absolute ethanol corresponding to the volume used in the series with the maximum concentration was used.

To determine the efficacy of these different concentrations, the sampling for AVC and planktonic cell count was performed in 3 different samples for controls and each concentration at 4, 24 and 48 h as described in Section 2.4.1 and Section 2.4.2, respectively.

#### 2.5.3. Epifluorescence Microscopy Assays

So as to obtain a clearer picture of the effects of the Fn1 and Fn3 dosed at different concentrations, samples were stained using FilmTracer^TM^ LIVE/DEAD^®^ Biofilm Viability Kit (Life Technologies, Eugene, OR, USA) as described elsewhere [34]. This stain allows undamaged/live and damaged/dead cells based on their membrane integrity fluorescing in green or red, respectively, to be distinguished. After preparation, visualisation of the samples was performed using a Leica 6000DM (Leica, Wetzlar, Germany) epifluorescence microscope using a 40× objective and 10× ocular lenses coupled with a Leica DFC365 FX camera. Image acquisition was carried out using Metamorph MMAF software (Molecular Devices, Sunnyvale, CA, USA).

#### 2.5.4. Effects of Fn3 Dosed at Different Times of L34 Biofilm Maturation

In this phase, the inhibitory effects of Fn3 at different stages of maturation on *L. monocytogenes* L34 biofilms were tested. Biofilms were cultured as described in Section 2.3. Following this, at each dosage time, Fn3 was added to samples at a concentration of 20 mmol L^−1^ as follows:

- First, an addition of Fn3 to 9 coupons at *t* = 0 h with subsequent AVC sampling at 24, 48 and 72 h (*n* = 3 coupons per sampling time). This triple sampling was intended to better monitor the capability of Fn3 to inhibit the initial biofilm formation and the effects of a single dosage after 72 h.
- Second, an addition of Fn3 to 3 coupons at *t* = 24 h with subsequent AVC sampling at 96. In this case, the sampling was intended to ascertain the antibiofilm effect of Fn3 on *L. monocytogenes* L34 early-stage biofilm by an end-point measurement 72 h after the furanone was added to the culture.
- Finally, an addition of Fn3 to 3 coupons at *t* = 96 h followed by subsequent AVC sampling at 168 h. On this occasion, the assay was focused on the determination of the biofilm inhibitory effects of Fn3 applied on mature structures also by an end-point sampling 72 h after the addition of the furanone.

In all cases, for control samples (*n* = 3 per each sampling time), an equal volume of absolute ethanol was added to each well to discard the individual effects of the solvent on the biofilm. Sampling and quantification of AVC cells were performed following the protocol described in Section 2.4.1.

### 2.6. Statistical Analysis

Two different statistical tests were performed to assess significance among the obtained results using IBM SPSS Statistics for Windows, Version 23.0 (IBM Corp., Armonk, NY, USA).

Specifically, to statistically compare the AVC values in each individual isolate at 24 and 168 h, a two-tailed Student’s *t*-test was used. Next, a two-way ANOVA with an HSD Tukey’s post hoc test was chosen to determine statistically significant differences of biofilm formation among all the isolates at 24 and 168 h and also to assess significance in the level of adhesion regarding the sources.

Following this, a two-tailed Student’s *t*-test was preferred to determine statistical differences in the first furanones’ efficacy screening (Section 2.5.1.) and in the additional experiments performed with Fn1 and Fn3 (Section 2.5.2.).

Finally, a paired two-tailed Student’s *t*-test was chosen to assess significance between the level of adhesion at each sampling time of the assays involving the addition of furanones at different biofilm age (Section 2.5.4.).

In all cases, significance was expressed at the 95% confidence level (*α* = 0.05) or greater.

## 3. Results

### 3.1. Subtyping

**Figure 1:**
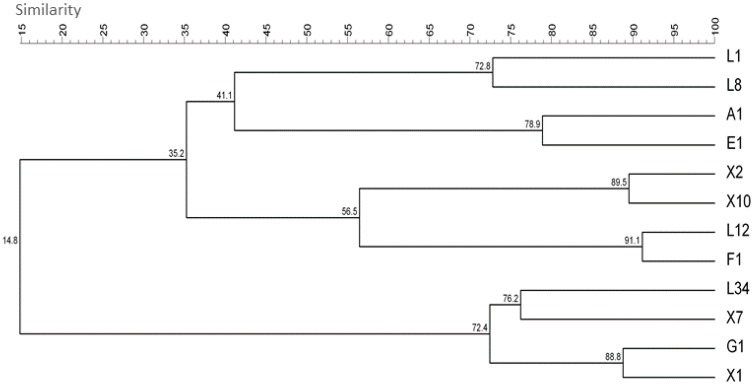
*Listeria monocytogenes* isolates dendrogram corresponding to the Unweighted Pair Group Method with Arithmetic mean (UPGMA) cluster analysis obtained after combining the OMP01, DAF4, HLWL85 and UBC155 random-amplified polymorphic DNA (RAPD)-PCR band patterns.

The subtype of the different isolates of *L. monocytogenes* was determined by combining the band patterns obtained in the four RAPD-PCR assays. As depicted in Figure 1, composite dendrograms based on the UPGMA cluster analysis showed that no relationship could be established among the isolates assayed, either between isolates forming a determined sector or between sectors (discrimination index = 0.99; [35]). Taking a closer look at the dendrogram, it can be observed that the two main clusters are composed of at least one clinical isolate, one from foodstuffs and one from food-related surfaces, further confirming the above mentioned.

### 3.2. AVC Quantification in Early- and Late-Stage Biofilms on SS

To observe if there was any difference in the adhesion capability of the *L. monocytogenes* isolates obtained from different sources (i.e., foodstuffs, environmental and clinical), biofilms were grown onto AISI 316 SS surfaces, and the number of adhered viable and cultivable (AVC) cells was determined after 24 and 168 h.

**Figure 2:**
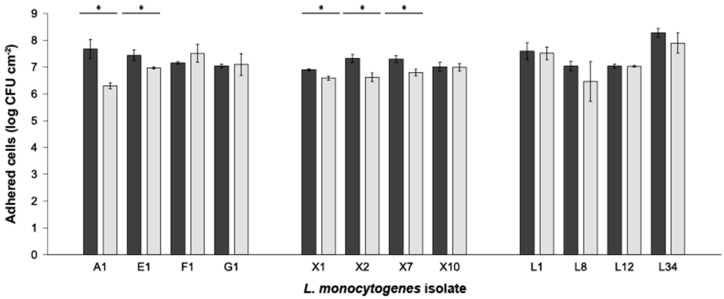
Adhered viable and cultivable (AVC) *L. monocytogenes* cells on AISI 316 stainless steel coupons after 24 (dark bars) and 168 h (light bars). Bars indicate the mean value (*n* = 3) expressed in log CFU cm^−2^. Error bars represent the standard deviation of each group of samples. Asterisks indicate significant differences between the level of adhesion between 24 and 168 (two-tailed Student’s *t*-test; *α* = 0.05). Additionally, a two-way ANOVA (*α* = 0.05) was performed to assess the significance between the mean value of all the isolates at 24 and 168 h. For details regarding the latter, the reader is kindly referred to the text (Section 3.2). For a complete output of the two-way ANOVA, the reader is referred to Supplementary Tables S1 and S2.

If compared individually, strains A1 and E1 (environmental sources), and X1, X2 and X7 (clinical sources) presented AVC significance (two-tailed Student’s *t-*test; *p* < 0.05) with lower counts at 168 h compared to those obtained at 24 h (Figure 2). Outcomes also showed, generally, no differences (two-way ANOVA; *p* > 0.05) in the level of adhesion among the strains assayed (Figure 2, Supplementary Table S1). Of note, the L34 strain presented the highest significant differences (*p* < 0.05) compared with all the other strains tested but with A1 in the amount of *L. monocytogenes* AVC at 24 h and with all but with strains F1, G1, L1 and L12 at 168 h (Supplementary Table S1). Considering the strains’ sources, statistically significant differences (*p* < 0.05) were obtained between clinical compared to environmental and food strains at 24 h. Contrarily, at 168 h, significance (*p* < 0.05) was only observed between clinical and food sources (Supplementary Tables S1 and S2).

For a complete detail of the data regarding the two-way ANOVA analysis, the reader is kindly referred to Supplementary Tables S1 and S2, displaying comparing the differences in AVC counts between strains at 24 and 168, respectively. Thus, considering the levels of adhesion obtained and for further experimentation, strains F1, X10 and L34 were chosen as representatives of environmental, clinical and food strains, respectively.

### 3.3. Effect of Synthetic Furanones on L. monocytogenes Adhesion onto SS Coupons

The effects of six molecularly different furanones were tested to determine the inhibition of biofilm formation at 24 h of three *L. monocytogenes* strains coming from different sources.

**Figure 3:**
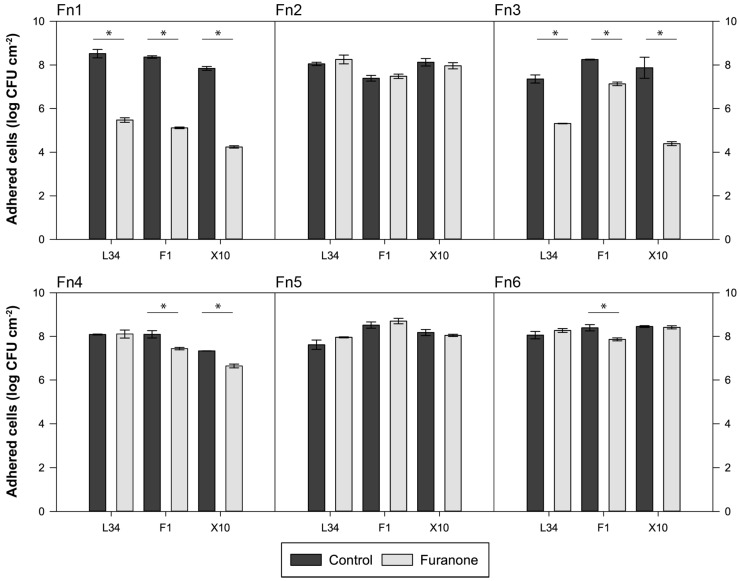
Effect of synthetic furanones on the formation of *Listeria monocytogenes* 24 h biofilms onto stainless steel coupons. Bars represent the mean value (*n* = 3) expressed in log CFU cm^−2^ of adhered viable and cultivable cells harvested at 24 h in after the addition of 2 mmol L^−1^ (Fn1) or 20 mmol L^−1^ (Fn2 to Fn6) furanone with respect to the control. Error bars represent the standard deviation of each group of samples. Asterisks indicate significance between series (two-tailed *t*-test; *α* = 0.05).

As depicted in Figure 3, results showed how halogenated furanones, Fn1 (brominated) and Fn3 (chlorinated), produced a decrease in the level of AVC at 24 h in all three strains tested. In both cases, *L. monocytogenes* X10 was the most sensitive strain to both compounds with a reduction of 3.60 and 3.46 log CFU cm^−2^ for Fn1 and Fn3, respectively (Figure 3). On the other side, Fn4 and F6 produced a more discreet AVC reduction in strains F1 (Fn4 and Fn6) and X10 (only Fn4) (Figure 3). Of note, strain L34 adhesion was not affected by any other furanone but Fn1 and Fn3.

Once the effects of the different furanones were determined and based on the maximum reductions of AVC counts obtained, Fn1 and Fn3, i.e., halogenated furanones, were chosen to evaluate their individual effects at different concentrations and dosage at various maturation stages on *L. monocytogenes* L34 biofilms in terms of adhesion and maturation, and planktonic cell growth.

### 3.4. Effect of Halogenated Furanones on L34 on Adhesion and Planktonic Cell Growth

As a first complementary experiment to assess individual efficacy of the furanones against L34 biofilm formation, Fn1 and Fn3 were dosed at different concentrations at 0 h, and their effects on adhered and planktonic cells were assessed at 4, 24 and 48 h.

**Figure 4:**
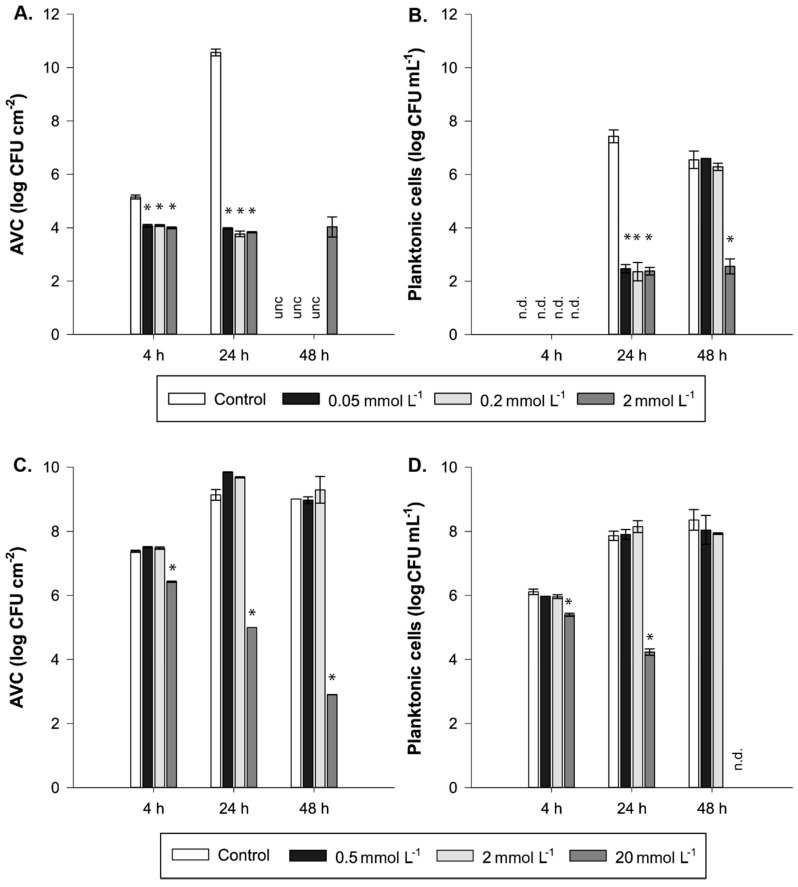
Effects of dosage of different concentrations on Fn1 (**A**,**B**) and Fn3 (**C**,**D**) on the number of AVC cells and planktonic cells in *L. monocytogenes* L34 biofilm cultures at 4, 24 and 48 h. Bars indicate the mean value of *n* = 3 coupons, and the error bars depict the standard deviation of each sample set. Asterisks indicate the statistical significance of the value compared with the control (two-tailed *t-*test; *α* = 0.05). unc; uncountable plate count, n.d.: not determined.

As portrayed in Figure 4A, it can be observed that regardless of the Fn1 concentration, the adhesion of L34 cells was impaired up to 24 h with a mean difference of 6.71 log CFU cm^−2^ regarding the control. Contrarily, at 48 h, only the highest furanone concentration used (2 mmol L^−1^) was able to maintain this anti-biofilm effect (Figure 4A).

**Figure 5:**
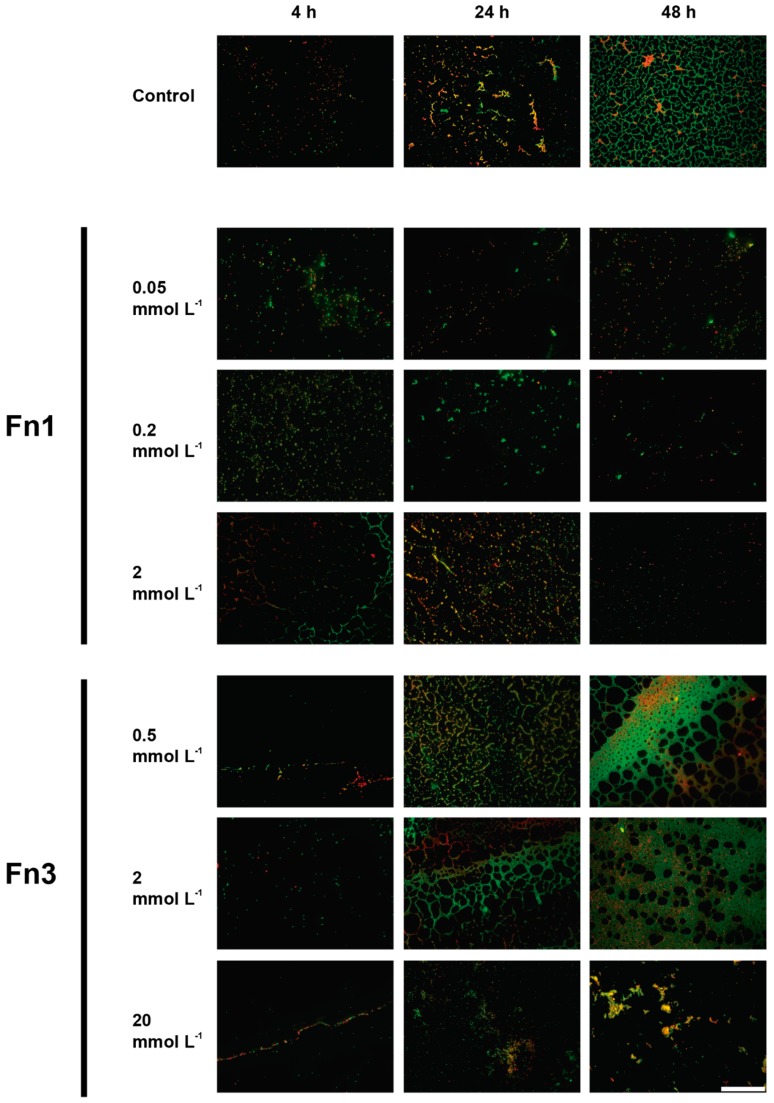
Representative epifluorescence 40× field micrographs corresponding to 4, 24 and 48 h-old *L. monocytogenes* L34 biofilms grown on AISI 316 stainless steel coupons after the addition of Fn1 and Fn3 at different concentrations. Samples were treated with LIVE/DEAD staining. Green-emitting cells represent undamaged (live) cells, and red-emitting cells represent those either injured or dead. Scale bar = 50 μm.

*De visu* analysis of microscopy images was in line with these results (Figure 5). As observed, the cellular density in furanone-treated samples was significantly low compared with the control samples. It is also important to remark that even though the number of adhered cells present in the samples was lower, among those still present, there was no significant increase in the amount of red (damaged) cells (Figure 5).

Furthermore, the number of planktonic cells at 24 h in Fn1-treated samples is about 5 log CFU cm^−2^ lower than controls. On the contrary, this effect at 48 h was present in samples where the highest concentration of furanone (i.e., 2 mmol L^−1^) was used, with a difference of 3.99 log CFU mL^−1^ compared with controls. At concentrations 0.2 and 0.05 mmol L^−1^, no significance was observed either between them or compared with control samples.

In the case of Fn3, significant differences were only present at the highest concentration used (i.e., 20 mmol L^−1^). Results demonstrate how the AVC counts were lowered in all sampling times with a difference of 0.71 and 3.64 log CFU cm^−2^ at 4 and 24 h, respectively (Figure 4C,D). Epifluorescence microscopy images further confirmed these results (Figure 5). As observed, the morphology in 20 mmol L^−1^ treated sample was severely affected, especially at 48 h in which the number of adhered cells, both green and red-emitting, was hindered. Moreover, the architecture of the biofilm appeared visibly altered with few aggregates present compared with the control in which a dense, uniformly distributed biofilm predominated (Figure 5). Of note, at lower concentrations of the furanone used (i.e., 0.5 and 2 mmol L^−1^), a visible alteration of the structure of the 48 h biofilm was present in which the structured covered more area compared to control (Figure 5).

### 3.5. Effects of F3 Early- and Mid-Term Dosage on L34 Biofilm Development

To get a clearer picture of the long-term effects of Fn3, this was tested in *L. monocytogenes* L34 biofilms at different maturation stages. The furanone was dosed at 0, 24, and 96 h of biofilm growth, and the AVC counts were determined at different times. Specifically, when dosed at t0, a triple sampling (i.e., 24, 48 and 72 h after the addition of Fn3) was performed to ascertain the capability of the furanone to prevent adhesion at early stages. In the other two test groups, an end-point sampling (i.e., 72 h after the addition of the furanone) was preferred to determine if the compound was able to dwindle the AVC count onto the SS surface on more mature structures.

**Figure 6:**
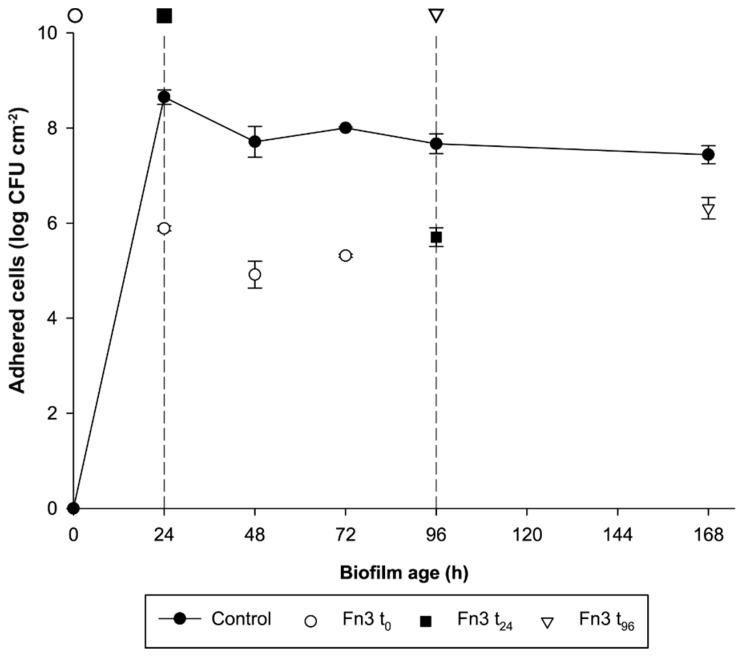
Effects, expressed in log CFU cm^−2^, of Fn3 dosed at different maturation stages on *Listeria monocytogenes* L34 biofilms. Reference lines and symbols on the top of the graph at 0 (○), 24 (■) and 96 h (∇) indicate the moments in which Fn3 was dosed on non-treated biofilms. Then, the same symbols in the graph correspond to the AVC counts regarding the previous furanone addition (see Materials and Methods for further details on the furanone dosage schemes followed). Each value represents the mean of *n* = 3 samples (i.e., stainless steel coupons). Error bars indicate the standard deviation. All values of furanone-treated samples were significantly different from the control (two-tailed *t*-test; *α* = 0.05).

As can be observed in Figure 6, in all cases, the amount of AVC cells in the furanone-treated biofilm was significantly different (two-tailed *t*-test; *α* = 0.05) with respect to the control. The biggest differences in AVC cells were obtained at 24, 48 and 72 h (Fn3 added at 0 h) with differences compared to controls of 2.76, 2.79 and 2.68 log CFU cm^−2^, respectively (Figure 6).

Counts at 96 and 168 h (i.e., previous Fn3 addition at 24 and 96 h, respectively) gave a difference of 1.96 log and 1.12 log CFU cm^−2^, respectively, in treated biofilms regarding controls (Figure 6). This indicated that further furanone addition, despite being still effective, produced a more discreet effect on AVC cell count and that its antibiofilm efficacy decreased as the addition took place at more advanced stages of biofilm development.

## 4. Discussion

*Listeria monocytogenes* is a pathogen of serious concern with an increasing incidence trend among the human population [4]. Among strains tested, and regardless of the subtype, there were no differences among the levels of AVC on SS (Figure 2) in concordance with previously published data showing no differences among different *L. monocytogenes* strains [28] but in contrast to previous studies demonstrating that biofilm formation is strain-specific [36,37,38]. Such controversy can be attributed to the experimental design based on plate count that does not permit the identification of dynamic differences as other methods, such as microscopy plus image analysis, do [39,40].

Halogenated furanones have been described as efficient biofilm inhibitors in various Gram-negative [21,22,41] and Gram-positive bacteria [42,43], however, to the best of our knowledge, there are no previous studies testing halogenated furanones on *L. monocytogenes*. Nonetheless, studies involving food-related spoilage organisms, such as the one carried out by Ren et al., demonstrated the efficacy of Fn1 on *Bacillus subtilis* biofilms, reducing the amount of biomass formed on stainless steel and also its planktonic growth, being thus in line with the outcomes presented (Figure 3) [11]. In addition, in *B. subtilis*, the efficacy of mucobromic and mucochloric acids, analogues to Fn1 and Fn3, respectively with a hydroxyl group in C5, have proven to inhibit biofilm formation preventing its adherence in polystyrene plates [23]. Moreover, brominated furanones have been proven to interfere with AI-2 systems [20,43,44,45]. Hence, since *L. monocytogenes* is able to secrete and responds to functional AI-2-like molecules [10,46], such effects of Fn1 and Fn3 could have been a consequence of inhibition of the quorum sensing pathway.

In Gram-positives, Fn1 has been demonstrated to have antibiofilm capacity, as shown by the hereby presented outcomes (Figure 3, Figure 4 and Figure 5). As a matter of example, the study carried out by He et al. demonstrated that the addition of either 2 or 4 µg mL^−1^ Fn1 impaired the biofilm formation in *Streptococcus mutans* and also that these effects were related with the downregulation of QS-mediated and environmental sensing genes [47]. In environmental studies, such as the one carried out by Zhao et al., observed a significant decrease in biofilm biomass production in *Acidithiobacillus ferrooxidans* [48]. In food systems, Fn1 has been demonstrated to be efficient against food spoilage in fresh produce [49]. Hence, in light of the results presented in this article, the usage of Fn1 on food-related surfaces to avoid biofilm colonisation seems a feasible alternative to be explored.

Nevertheless, it is important to consider that in the experiments performed with Fn1, 48 h AVC counts corresponding to controls and those corresponding to series treated with 0.2 and 0.05 mmol L^−1^ of furanone were above the limit of detection, i.e., too many colonies to be counted individually (Figure 4A). Consequently, it was not possible to determine if there were still differences in these two latter series to the control. Nonetheless, if we observe results regarding planktonic cells in which only the highest concentration was effective (Figure 4B), it is logical to think that higher counts were obtained in both the control and those corresponding to the lowest concentrations. Of note, among treated series, it cannot be assumed that counts were equal to the control since there was a delay in the adhesion of cells as observed (Figure 4A), so differences could still be present.

Although it is generally accepted that synthetic furanones do not affect planktonic growth [18], outcomes obtained in this study demonstrate that even at low concentrations, growth in *L. monocytogenes* L34 is impaired by Fn1 and Fn3 (Figure 4). This fact is in discordance with previously reported data involving Gram-positives, such as *S. mutans* [47] or *A. ferrooxidans* [48], in which the addition of halogenated furanones did not affect the planktonic growth of either species, even though the expression of genes involved in biofilm formation was downregulated.

Regarding Fn3, biofilm morphology was visually altered when a concentration of 20 mmol L^−1^ was used whilst, at lower concentrations, the surfaced covered by the green-emitting (live) cells seemed to be higher (Figure 5). Nonetheless, if the AVC counts (Figure 4C) are considered, it can be observed that not statically significant differences were present (two-tailed *t*-test; *α* = 0.05). Hence, since epifluorescence microscope image acquisition lacks on the third dimension (thickness), it could be hypothesised that the number of cells present in the sample was the same but in a different tri-dimensional configuration, i.e., the same number of cells but more distributed in a thinner biofilm.

As depicted in Figure 6, chlorinated furanone (i.e., Fn3) significantly reduced the amount of AVC when applied at different stages of biofilm maturation (i.e., when dosed at 0, 24 and 96 h). The results obtained in the present study are in line with previously published data demonstrating the biofilm inhibitory effects of such compounds on the early stages of biofilm formation in different species [50,51,52,53].

Similar inhibitory effects have been previously observed in Gram-positives, such as *B. subtilis,* biofilms grown onto stainless steel and treated with halogenated furanones, with an effect that lasted at for least 48 h [11]. To the best of the authors’ knowledge, no studies have been found using Fn3 against bacterial biofilms. Nonetheless, chemical analogues to Fn3, such as mucochloric acid and other derivatives, have been found to have an effect against early biofilm formation of *B. subtilis* [23], *Streptococcus mutans* [54] and *Pseudomonas aeruginosa* [55], among others. Nevertheless, this is the first report testing the effects of different dosages along time of 3,4-dichloro-2(5*H*)-furanone against *L. monocytogenes* biofilms.

## 5. Conclusions

The outcomes obtained in this study demonstrated that the addition of furanones, especially those with halogen groups, to the culture medium is able to significantly inhibit the planktonic growth and the adhesion of *L. monocytogenes* to stainless steel surfaces. However, such effects are sharper within the early stages of biofilm development and are dose-dependent. Moreover, it was demonstrated that the efficacy of these compounds decrease as the biofilm becomes more mature.

Even though further experimentation is required to ascertain the actual effects of Fn1 and Fn3 on the quorum sensing pathways, results obtained in this work can be used as a first approach to develop novel, environmentally friendly biofilm control systems, thus making synthetic furanones a feasible strategy to prevent *L. monocytogenes* biofilm formation.

## Figures and Tables

**Table 1:** Codes and origins of the *Listeria monocytogenes* isolates used in this study.

Source	Code	Origin	Reference
Environmental	A1	Thermal gloves	[28]
	E1	Transportation trolley	[28]
	F1	Meat mincer	[28]
	G1	Milking device	[29]
Food	L1	Crab salad	[30]
	L8	Deli tuna salad	[30]
	L12	Halibut fillet	This study
	L34	Frozen panga fillet	[30]
Clinical	X1	Human listeriosis	This study
	X2	Human listeriosis	This study
	X3	Human listeriosis	This study
	X7	Human listeriosis	This study
	X10	Human listeriosis	This study

**Table 2:** Synthetic furanones used in this study with their corresponding codes used throughout the manuscript.

Code	Furanone Name (Synonym)	Empirical Formula	Structure
Fn1	(Z-)-4-Bromo-5-(bromomethylene)-2(5*H*)-furanone (Furanone C-30)	C_5_H_2_Br_2_O_2_	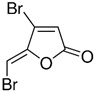
Fn2	2-Methyltetrahydro-3-furanone	C_5_H_8_O_2_	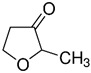
Fn3	3,4-Dichloro-2(5H)-furanone	C_4_H_2_Cl_2_O_2_	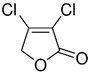
Fn4	4-Hydroxy-2,5-dimethyl-3(2*H*)-furanone (Furaneol)	C_6_H_8_O_3_	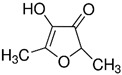
Fn5	Dihydro-3-amino-2-(3*H*)-furanone	C_4_H_7_NO_2_	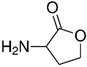
Fn6	(*S*)-(+)-Dihydro-5-(hydroxymethyl)-2(3*H*)-furanone	C_5_H_8_O_3_	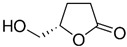

## Supplementary Materials

The following are available online at https://www.mdpi.com/2304-8158/8/12/647/s1, Table S1: Complete output of the two-way ANOVA analysis of the AVC counts at 24h, Table S2: Complete output of the two way ANOVA analysis of the AVC counts at 168 h.
