# Supplementary material for: Efficacy of Synthetic Furanones on Listeria monocytogenes Biofilm Formation
Source: Foods. 2019 Dec 5;8(12):647. doi: 10.3390/foods8120647 (PMC6963563; doi:10.3390/foods8120647)
Supplement: Supplementary file 1 [file foods-08-00647-s001.pdf]

TWO WAY ANOVA OUTPUT (168 h)

Table S1. Multiple comparisons (AVC).

(I) Isolate	(J) Isolate	Mean difference (I-J)	SE	Sign.	Confidence interval 95%	
					Lower limit	Upperlimit
A1	E1	0.2322	0.15170	0.917	-0.3148	0.7791
	F1	0.5195	0.15170	0.073	-0.0274	1.0665
	G1	,6271*	0.15170	0.015	0.0802	1.1741
	X1	,7753*	0.15170	0.002	0.2284	1.3223
	X2	0.3501	0.15170	0.497	-0.1969	0.8971
	X7	0.3759	0.15170	0.397	-0.1711	0.9228
	X10	,6659*	0.15170	0.008	0.1190	1.2129
	L1	0.0845	0.15170	1.000	-0.4625	0.6314
	L8	,6410*	0.15170	0.012	0.0940	1.1879
	L12	,6404*	0.15170	0.013	0.0934	1.1874
	L34	-,5588*	0.15170	0.042	-1.1058	-0.0119
E1	A1	-0.2322	0.15170	0.917	-0.7791	0.3148
	F1	0.2874	0.15170	0.752	-0.2596	0.8343
	G1	0.3949	0.15170	0.330	-0.1520	0.9419
	X1	0.5432	0.15170	0.053	-0.0038	1.0901
	X2	0.1179	0.15170	1.000	-0.4290	0.6649
	X7	0.1437	0.15170	0.998	-0.4033	0.6907
	X10	0.4338	0.15170	0.217	-0.1132	0.9807
	L1	-0.1477	0.15170	0.997	-0.6947	0.3993
	L8	0.4088	0.15170	0.286	-0.1382	0.9558
	L12	0.4082	0.15170	0.288	-0.1387	0.9552
	L34	-,7910*	0.15170	0.001	-1.3380	-0.2440
F1	A1	-0.5195	0.15170	0.073	-1.0665	0.0274
	E1	-0.2874	0.15170	0.752	-0.8343	0.2596
	G1	0.1076	0.15170	1.000	-0.4394	0.6546
	X1	0.2558	0.15170	0.858	-0.2911	0.8028
	X2	-0.1694	0.15170	0.991	-0.7164	0.3775
	X7	-0.1437	0.15170	0.998	-0.6906	0.4033
	X10	0.1464	0.15170	0.997	-0.4006	0.6934
	L1	-0.4351	0.15170	0.214	-0.9820	0.1119
	L8	0.1214	0.15170	0.999	-0.4255	0.6684
	L12	0.1209	0.15170	0.999	-0.4261	0.6678
	L34	-1,0784*	0.15170	0.000	-1.6253	-0.5314
G1	A1	-,6271*	0.15170	0.015	-1.1741	-0.0802
	E1	-0.3949	0.15170	0.330	-0.9419	0.1520
	F1	-0.1076	0.15170	1.000	-0.6546	0.4394
	X1	0.1482	0.15170	0.997	-0.3987	0.6952
	X2	-0.2770	0.15170	0.789	-0.8240	0.2699
	X7	-0.2513	0.15170	0.871	-0.7982	0.2957
	X10	0.0388	0.15170	1.000	-0.5082	0.5858
	L1	-0.5427	0.15170	0.053	-1.0896	0.0043
	L8	0.0138	0.15170	1.000	-0.5331	0.5608
	L12	0.0133	0.15170	1.000	-0.5337	0.5602
	L34	-1,1860*	0.15170	0.000	-1.7329	-0.6390
X1	A1	-,7753*	0.15170	0.002	-1.3223	-0.2284
	E1	-0.5432	0.15170	0.053	-1.0901	0.0038
	F1	-0.2558	0.15170	0.858	-0.8028	0.2911
	G1	-0.1482	0.15170	0.997	-0.6952	0.3987

	X2	-0.4252	0.15170	0.239	-0.9722	0.1217
	X7	-0.3995	0.15170	0.315	-0.9464	0.1475
	X10	-0.1094	0.15170	1.000	-0.6564	0.4376
	L1	-,6909*	0.15170	0.006	-1.2378	-0.1439
	L8	-0.1344	0.15170	0.999	-0.6813	0.4126
	L12	-0.1349	0.15170	0.999	-0.6819	0.4120
	L34	-1,3342*	0.15170	0.000	-1.8811	-0.7872
X2	A1	-0.3501	0.15170	0.497	-0.8971	0.1969
	E1	-0.1179	0.15170	1.000	-0.6649	0.4290
	F1	0.1694	0.15170	0.991	-0.3775	0.7164
	G1	0.2770	0.15170	0.789	-0.2699	0.8240
	X1	0.4252	0.15170	0.239	-0.1217	0.9722
	X7	0.0258	0.15170	1.000	-0.5212	0.5727
	X10	0.3158	0.15170	0.639	-0.2311	0.8628
	L1	-0.2656	0.15170	0.828	-0.8126	0.2813
	L8	0.2909	0.15170	0.738	-0.2561	0.8378
	L12	0.2903	0.15170	0.741	-0.2567	0.8373
	L34	-,9089*	0.15170	0.000	-1.4559	-0.3620
X7	A1	-0.3759	0.15170	0.397	-0.9228	0.1711
	E1	-0.1437	0.15170	0.998	-0.6907	0.4033
	F1	0.1437	0.15170	0.998	-0.4033	0.6906
	G1	0.2513	0.15170	0.871	-0.2957	0.7982
	X1	0.3995	0.15170	0.315	-0.1475	0.9464
	X2	-0.0258	0.15170	1.000	-0.5727	0.5212
	X10	0.2901	0.15170	0.741	-0.2569	0.8370
	L1	-0.2914	0.15170	0.736	-0.8384	0.2556
	L8	0.2651	0.15170	0.829	-0.2819	0.8121
	L12	0.2645	0.15170	0.831	-0.2824	0.8115
	L34	-,9347*	0.15170	0.000	-1.4817	-0.3877
X10	A1	-,6659*	0.15170	0.008	-1.2129	-0.1190
	E1	-0.4338	0.15170	0.217	-0.9807	0.1132
	F1	-0.1464	0.15170	0.997	-0.6934	0.4006
	G1	-0.0388	0.15170	1.000	-0.5858	0.5082
	X1	0.1094	0.15170	1.000	-0.4376	0.6564
	X2	-0.3158	0.15170	0.639	-0.8628	0.2311
	X7	-0.2901	0.15170	0.741	-0.8370	0.2569
	L1	-,5815*	0.15170	0.030	-1.1284	-0.0345
	L8	-0.0250	0.15170	1.000	-0.5719	0.5220
	L12	-0.0255	0.15170	1.000	-0.5725	0.5214
	L34	-1,2248*	0.15170	0.000	-1.7717	-0.6778
L1	A1	-0.0845	0.15170	1.000	-0.6314	0.4625
	E1	0.1477	0.15170	0.997	-0.3993	0.6947
	F1	0.4351	0.15170	0.214	-0.1119	0.9820
	G1	0.5427	0.15170	0.053	-0.0043	1.0896
	X1	,6909*	0.15170	0.006	0.1439	1.2378
	X2	0.2656	0.15170	0.828	-0.2813	0.8126
	X7	0.2914	0.15170	0.736	-0.2556	0.8384
	X10	,5815*	0.15170	0.030	0.0345	1.1284
	L8	,5565*	0.15170	0.044	0.0095	1.1035
	L12	,5559*	0.15170	0.044	0.0090	1.1029
	L34	-,6433*	0.15170	0.012	-1.1903	-0.0963
L8	A1	-,6410*	0.15170	0.012	-1.1879	-0.0940
	E1	-0.4088	0.15170	0.286	-0.9558	0.1382
	F1	-0.1214	0.15170	0.999	-0.6684	0.4255
	G1	-0.0138	0.15170	1.000	-0.5608	0.5331
	X1	0.1344	0.15170	0.999	-0.4126	0.6813

	X2	-0.2909	0.15170	0.738	-0.8378	0.2561
	X7	-0.2651	0.15170	0.829	-0.8121	0.2819
	X10	0.0250	0.15170	1.000	-0.5220	0.5719
	L1	-,5565*	0.15170	0.044	-1.1035	-0.0095
	L12	-0.0006	0.15170	1.000	-0.5475	0.5464
	L34	-1,1998*	0.15170	0.000	-1.7468	-0.6528
L12	A1	-,6404*	0.15170	0.013	-1.1874	-0.0934
	E1	-0.4082	0.15170	0.288	-0.9552	0.1387
	F1	-0.1209	0.15170	0.999	-0.6678	0.4261
	G1	-0.0133	0.15170	1.000	-0.5602	0.5337
	X1	0.1349	0.15170	0.999	-0.4120	0.6819
	X2	-0.2903	0.15170	0.741	-0.8373	0.2567
	X7	-0.2645	0.15170	0.831	-0.8115	0.2824
	X10	0.0255	0.15170	1.000	-0.5214	0.5725
	L1	-,5559*	0.15170	0.044	-1.1029	-0.0090
	L8	0.0006	0.15170	1.000	-0.5464	0.5475
	L34	-1,1992*	0.15170	0.000	-1.7462	-0.6523
L34	A1	,5588*	0.15170	0.042	0.0119	1.1058
	E1	,7910*	0.15170	0.001	0.2440	1.3380
	F1	1,0784*	0.15170	0.000	0.5314	1.6253
	G1	1,1860*	0.15170	0.000	0.6390	1.7329
	X1	1,3342*	0.15170	0.000	0.7872	1.8811
	X2	,9089*	0.15170	0.000	0.3620	1.4559
	X7	,9347*	0.15170	0.000	0.3877	1.4817
	X10	1,2248*	0.15170	0.000	0.6778	1.7717
	L1	,6433*	0.15170	0.012	0.0963	1.1903
	L8	1,1998*	0.15170	0.000	0.6528	1.7468
	L12	1,1992*	0.15170	0.000	0.6523	1.7462

Dependent variable: Adhered cells. Tukey's HSD. Based on observed mean. The error term is quadratic mean(error) = 0.035. * Significant differences at level 0,05.

Table 2. Multiple comparisons (Source).

**(I) Source**	**(J) Source**	**Mean difference (I-J)**	**SE**	Sign.	Confidence interval 95%	
					Lower limit	Upper limit
**Envrionmental**	**Clinical**	**,1971***	**0.07585**	0.040	0.0077	0.3865
	Food	-0.1430	0.07585	0.165	-0.3324	0.0465
Clinical	Environmental	-,1971*	0.07585	0.040	-0.3865	-0.0077
	Food	-,3401*	0.07585	0.000	-0.5295	-0.1506
Food	Environmental	0.1430	0.07585	0.165	-0.0465	0.3324
	Clinical	,3401*	0.07585	0.000	0.1506	0.5295

Dependent variable: Adhered cells. Tukey's HSD. Based on observed mean. The error term is quadratic mean(error) = 0.035. * Significant differences at level 0,05.
